# Predictive Role of Moral Injury and Burnout in Professional Communication: A Cross‐Sectional Study Among Operating Room Nurses

**DOI:** 10.1155/jonm/4905589

**Published:** 2026-07-29

**Authors:** Amirali Alizadeh, Zahra Maleki, Mahdiyeh Shahmoradi, Fatemeh Davodabadi, Fatemeh Vizeshfar

**Affiliations:** ^1^ Research Center for Evidence-Based Health Management, Maragheh University of Medical Sciences, Maragheh, Iran, mrgums.ac.ir; ^2^ Health Sciences Research Center, Torbat Heydariyeh University of Medical Sciences, Torbat Heydariyeh, Iran, thums.ac.ir; ^3^ Student Research Committee, Nursing and Midwifery College, Shiraz University of Medical Sciences, Shiraz, Iran, sums.ac.ir; ^4^ Student Research Committee, Hamadan University of Medical Sciences, Hamadan, Iran, umsha.ac.ir; ^5^ Community Based Psychiatric Care Research Center, Department of Nursing, School of Nursing and Midwifery, Shiraz University of Medical Sciences, Shiraz, Iran, sums.ac.ir

**Keywords:** burnout, moral injury, operating room, perioperative nurses, professional communication

## Abstract

**Aim(s):**

This study examined the association of moral injury and burnout with professional communication among operating room (OR) nurses in Southern Iran.

**Background:**

Effective professional communication is essential for surgical teamwork and patient safety. However, limited context‐specific evidence is available on how moral injury and burnout are jointly associated with the quality of professional communication among OR nurses. Addressing this gap may help inform strategies to improve staff well‐being, team functioning, and patient safety in high‐stakes perioperative settings.

**Methods:**

A cross‐sectional analytical study was conducted between March and May 2025. A total of 307 OR nurses were recruited from six tertiary referral hospitals in Shiraz, Iran. Data were collected using a demographic questionnaire, the Moral Injury Symptom Scale–Healthcare Professionals (MISS‐HP), the Operating Room Nurses’ Burnout Questionnaire, and the Professional Communication Questionnaire for Operating Room Personnel. Data were analyzed using descriptive and inferential statistics.

**Results:**

Moral injury was significantly and positively correlated with burnout. In the multivariate regression model, moral injury was positively associated with professional communication, whereas burnout showed a negative association.

**Conclusion:**

Moral injury and burnout were associated with professional communication among OR nurses. While burnout was negatively associated with communication, the unexpected positive adjusted association between moral injury and professional communication should be interpreted cautiously and may reflect ethically motivated communication, moral sensitivity, or professional advocacy rather than a beneficial effect of moral injury itself.

**Implications for Nursing Management:**

Nursing managers should consider integrated strategies to reduce burnout, support nurses facing morally challenging situations, and strengthen professional communication within surgical teams. Practical approaches may include workload monitoring, fair shift allocation, moral resilience training, ethics debriefing, supportive supervision, and team‐based communication training. Such interventions may help promote nurses’ psychological well‐being while supporting safer and more effective communication in OR settings.

## 1. Introduction

The operating room (OR) is one of the most complex and high‐stress environments in modern healthcare. It requires time‐critical decision‐making, seamless interdisciplinary collaboration, and strict adherence to patient safety protocols [1]. These elements are closely linked to treatment outcomes and depend heavily on effective professional communication. As integral members of the surgical team, perioperative nurses perform a wide range of responsibilities, including assisting surgeons, coordinating intraoperative care, maintaining an aseptic technique, and supporting patient safety throughout the surgical process. However, these responsibilities are often carried out under considerable psychological, ethical, and occupational pressures arising from resource limitations, heavy workloads, and complex organizational structures [2, 3]. Consequently, the quality of communication in this dynamic setting is essential for patient safety, team coordination, and error prevention. Observational evidence indicates that communication failures occur in approximately 30% of team exchanges in the OR, with at least one‐third of these failures associated with risks to patient safety through increased cognitive load, interrupted routines, and heightened team tension [4].

## 2. Background

Within the OR, communication extends beyond the simple transfer of information. It includes verbal and nonverbal signals, timely feedback, collaborative decision‐making, and sustained coordination during high‐risk surgical procedures [5, 6]. Nurses are central to this process, as they are responsible not only for carrying out surgical directives but also for communicating critical patient information and maintaining team cohesion under pressure [7, 8]. However, this multidimensional communication process may be influenced by nurses’ psychological state, particularly when they encounter ethical dilemmas or prolonged emotional strain [9].

A key construct in this regard is moral injury, which in healthcare refers to the psychological harm that may occur when professionals are unable to act in accordance with their moral or ethical values because of systemic, organizational, or clinical constraints. Such experiences may evoke guilt, helplessness, frustration, and moral distress, potentially affecting collaboration and contributing to tension within clinical teams [10, 11]. Alongside moral injury, burnout—characterized by emotional exhaustion, depersonalization, and a reduced sense of personal accomplishment—remains a major challenge in demanding healthcare environments. Burnout has been linked to chronic occupational stress, lower professional satisfaction, increased risk of medical errors, and reduced engagement in workplace communication [12, 13].

Although moral injury and burnout have each been examined as important occupational and psychological phenomena, their relationship with professional communication in the OR context remains insufficiently understood. Recent studies have reported high levels of moral injury and burnout among nurses working in high‐pressure clinical environments [14, 15]. Nevertheless, limited context‐specific evidence is available on how these constructs are jointly associated with the quality of professional communication among OR nurses. This gap is particularly relevant in the Iranian healthcare context, where staff shortages, demanding shift schedules, and hierarchical team structures may intensify the pressures experienced by perioperative nurses. Therefore, examining moral injury and burnout as correlates of professional communication may provide useful evidence for nursing managers seeking to support staff well‐being and improve surgical team functioning.

### 2.1. The Study

This study examined the association of moral injury and burnout with the quality of professional communication among OR nurses in Southern Iran. Using a cross‐sectional analytical design, the study addressed the following research question:

**Research Question:** *How are moral injury and burnout associated with professional communication among operating room nurses?*

**Hypotheses:** Based on the literature and theoretical background, we hypothesized that (1) moral injury and burnout would be positively correlated; (2) higher burnout would be associated with poorer professional communication; and (3) demographic and occupational characteristics would be associated with professional communication.

## 3. Methods

### 3.1. Study Design

This study used a cross‐sectional analytical design and was conducted between March and May 2025. The study was designed and reported in accordance with the Strengthening the Reporting of Observational Studies in Epidemiology (STROBE) guidelines for observational studies [16].

### 3.2. Setting and Participants

The study was conducted in six major tertiary healthcare centers in Shiraz, which is recognized as the medical hub of southern Iran. These hospitals provide specialized and subspecialized surgical services and manage a high volume of elective and emergency surgical cases. The target population consisted of nurses working in the OR units of these centers.

Participants were eligible for inclusion if they met the following criteria: (1) holding at least an associate degree in nursing or OR technology; (2) having a minimum of 6 months of continuous work experience in an OR; and (3) providing written informed consent to participate in the study.

Participants were excluded if they (1) were absent from the workplace during the data collection period because of leave, official assignment, or another authorized absence; (2) returned questionnaires with more than 10% missing responses; (3) reported ongoing psychiatric treatment; or (4) reported current use of medications affecting the central nervous system, including psychotropic or anxiolytic drugs.

### 3.3. Sample Size and Sampling Method

The sample size was calculated based on a previous study conducted by Zakeri–Afshar et al., using the formula for estimating a population mean. With a significance level (*α*) of 0.05, a standard deviation of 2.87, and a precision level of 0.4, the minimum required sample size was estimated to be 198 participants. Considering the cluster sampling design and applying a design effect of 1.5, the final target sample size was determined to be 307 nurses to ensure adequate statistical power.

Estimate a mean

(1) n≥ Z1−α/2×σ / d 2.

A multistage cluster sampling method was used. In the first stage, public hospitals affiliated with XXX University of Medical Sciences that had active OR units were purposively selected as the primary clusters. This purposive approach was used because these hospitals function as major teaching and referral centers in Southern XXX and provide a wide range of surgical workloads and specialties. Although the nonrandom selection of hospitals may limit generalizability, it was necessary to ensure sufficient sample availability and adequate representation of high‐acuity surgical settings.

In the next stage, with the collaboration of nursing managers in each center, a comprehensive list of OR nurses was compiled. Eligible participants were identified based on the inclusion and exclusion criteria. Each eligible nurse was assigned a unique code, and random sampling was performed using a random number table. The selected codes were then matched with the list, and the corresponding nurses were invited to participate in the study. This procedure helped minimize selection bias and supported the recruitment of a representative sample of OR nurses across the participating hospitals.

### 3.4. Data Collection Procedure

Data were collected in person across different work shifts, including morning, evening, and rotating shifts, by the principal researchers. This cross‐shift sampling strategy was used to reduce selection bias. Initially, the researchers visited the OR units of the selected hospitals, introduced themselves to the ward supervisors, and provided detailed explanations about the study objectives, significance, and intended use of the data. This process facilitated cooperation and created an appropriate context for voluntary participation.

Eligible nurses were then approached individually, and the study process was explained in detail. Participants received instructions on how to complete the questionnaires, were assured of anonymity and confidentiality, and were informed of their right to withdraw from the study at any stage without penalty. To reduce information bias and social desirability bias, which are important concerns in self‐report studies on sensitive topics such as moral injury, participants were assured that their responses would remain confidential and would have no effect on their professional evaluations or employment status. Written informed consent was obtained from all participants prior to enrollment.

### 3.5. Measurement Instruments

Data were collected using four instruments: a demographic and clinical characteristics questionnaire, the Moral Injury Symptom Scale–Healthcare Professionals (MISS‐HP), the Operating Room Nurses’ Burnout Questionnaire developed by Teymoori et al., and the Professional Communication Questionnaire for Operating Room Personnel developed by Torabizadeh et al.

The demographic and clinical characteristics questionnaire was developed by the research team and included parameters such as participants’ age, gender, marital status, number of monthly shifts, total working hours, and other job‐related variables relevant to OR nursing. The items were designed to align with the objectives and context of the present study.

Moral injury was assessed using the MISS‐HP, originally developed by Mantri et al. in 2020 [17] during the COVID‐19 pandemic [17]. This 10‐item scale uses a visual analog format ranging from 1 (*strongly disagree*) to 10 (*strongly agree*), yielding a total score between 10 and 100, with higher scores indicating greater moral injury. There are no reverse‐scored items in this scale. The MISS‐HP was translated and validated into Persian by Malakoutikhah al. [18], with minor adaptations to its dimensional structure. Whereas the original version assessed 10 domains—including betrayal, guilt, shame, moral concerns, loss of trust, loss of meaning or purpose, difficulty forgiving, blame, divine punishment, and loss of religious or spiritual faith—the Persian version evaluates eight domains, with two domains assessed by multiple items because overlapping constructs were merged during cross‐cultural psychometric adaptation. The Persian version also includes one additional item, excluded from the total score, which evaluates the perceived impact of moral injury on professional functioning. A cutoff score of 36.5 was established for the Persian version, compared with 36 in the original version. The psychometric properties of the scale have been confirmed in previous studies, with Cronbach’s alpha values of 0.75 for the original English version and 0.70 for the Persian validation study [18]. In the present study, Cronbach’s alpha for the MISS‐HP was 0.83, indicating good internal consistency.

Burnout was measured using the Operating Room Nurses’ Burnout Questionnaire, developed and validated in 2024 by Teymoori et al. [19] specifically for OR nurses. This 34‐item instrument assesses five dimensions: organizational structure, individual‐related factors, interpersonal factors, job characteristics, and managerial practices. Responses are rated on a five‐point Likert scale ranging from 0 (*never*) to 4 (*always*), producing a total score between 0 and 136. Higher scores indicate higher levels of occupational burnout. None of the items in this questionnaire are reverse‐scored. The instrument has demonstrated satisfactory validity and reliability, with an original Cronbach’s alpha of 0.93. In the current sample, Cronbach’s alpha for this questionnaire was 0.81, confirming good reliability.

Professional communication was evaluated using the Professional Communication Questionnaire for Operating Room Personnel, developed by Torabizadeh et al. in 2019. This 41‐item questionnaire assesses communication across six dimensions. Each item is scored on a five‐point Likert scale ranging from 1 (*never*) to 5 (*always*), with 13 items reverse‐scored, including items reflecting negative interactions or environmental barriers. The total score ranges from 93 to 153. Scores of 93–113 indicate poor communication, 114–133 indicate moderate communication, and 134–153 indicate strong professional communication. The instrument has been psychometrically validated, with an original Cronbach’s alpha of 0.88 [20, 20]. In the current study, Cronbach’s alpha for this questionnaire was 0.89, confirming its reliability in this sample.

### 3.6. Statistical Analysis

All analyses were performed using IBM SPSS Statistics, Version 27. Data were coded and entered into the software. The distribution of quantitative variables was assessed using the Kolmogorov–Smirnov test, Shapiro–Wilk test, histograms, Q–Q plots, and skewness and kurtosis indices. These assessments indicated that the data met the assumptions required for parametric analyses.

Incomplete questionnaires were handled using listwise deletion to maintain the integrity of the statistical models. Consequently, the final analysis was conducted on a complete dataset of 307 valid responses. Because the amount of missing data was minimal and no specific pattern of missingness was observed, no data imputation was performed.

Descriptive statistics, including mean, standard deviation, frequency, and percentage, were used to summarize participants’ demographic and study variables. Group comparisons were performed using independent‐samples *t*‐tests and one‐way analysis of variance (ANOVA), followed by Tukey’s post hoc tests where appropriate. Pearson’s correlation coefficients were used to examine bivariate associations among the main study variables. Finally, multiple linear regression analysis was conducted to examine the associations of demographic factors, moral injury, and burnout with professional communication.

### 3.7. Ethical Considerations

The study protocol was approved by the Biomedical Research Ethics Committee of Shiraz University of Medical Sciences (IR.SUMS.NUMIMG.REC.1403.114‎). All participants received oral and written information about the study objectives, procedures, and ethical safeguards. Written informed consent was obtained before data collection. Participation was entirely voluntary, and participants were assured that refusal to participate or withdrawal from the study would have no impact on their employment status.

To ensure confidentiality, each participant was assigned a unique nonidentifiable code, which was used throughout data entry, analysis, and reporting. No names or personal identifiers were recorded. All procedures were conducted in accordance with the principles of the Declaration of Helsinki, with respect for participants’ dignity, autonomy, and psychological safety [21].

## 4. Results

Of the eligible nurses approached across the 6 hospitals, 323 consented to participate. During data screening, the six incomplete cases were handled using listwise deletion, while the data of the 10 participants who withdrew consent were excluded from the analysis. Incomplete cases were handled using listwise deletion to maintain the integrity of the statistical models without relying on data imputation. The final sample therefore included 307 participants. The main reasons for nonparticipation were lack of time due to demanding work schedules and professional fatigue.

The mean age of participants was 31.48 ± 7.13 years. Overall, 42.7% were male and 57.3% were female. Additional demographic and occupational characteristics, including marital status, type of employment, educational attainment, work experience, shift type, monthly workload, and income distribution, are presented in Table 1.

**TABLE 1:** Demographic, occupational, and research variables of the participants (*N* = 297).

Variable	Category	*n* (%)	Mean ± SD
Age (years)	—	—	31.48 ± 7.13
Gender	Male	131 (42.7)	—
	Female	176 (57.3)	—

Marital status	Single	189 (61.6)	—
	Married	118 (38.4)	—

Employment type	Official	128 (41.7)	—
	Contractual	3 (1.0)	—
	Temporary	41 (13.4)	—
	Compulsory^∗^	135 (44.0)	—

Education level	Associate Degree	10 (3.3)	—
	Bachelor’s Degree	297 (96.7)^∗∗^	—

Work experience (years)	< 1 year	82 (26.7)	—
	1–5 years	92 (30.0)	—
	5–10 years	30 (9.8)	—
	> 10 years	103 (33.6)	—

Shift type	Morning	8 (2.6)	—
	Evening	8 (2.6)	—
	Rotating	291 (94.8)	—

Monthly shifts (number)	—	—	25.79 ± 7.89
Monthly scrub hours	—	—	27.55 ± 18.69
Monthly circulator hours	—	—	41.29 ± 30.79
Overtime hours (monthly)	—	—	52.17 ± 38.17
Income (million IRR^∗^)^∗∗^	< 10	3 (1.0)	—
	10–20	276 (89.9)	—
	> 20	28 (9.1)	—

Recent severe emotional trauma/loss (past 6 months)	Yes	75 (24.4)	—
	No	232 (75.6)	—

Workplace ethics training	Yes	47 (15.3)	—
	No	260 (84.7)	—

Main variables			
Moral Injury Score	—	—	41.12 ± 10.57
Job Burnout Score	—	—	90.43 ± 24.42
Professional Communication Score	—	—	129.58 ± 10.30
Professional communication level	Weak	13 (4.2%)	—
	Moderate	154 (50.2%)	—
	Strong	107 (34.9%)	—
	Missing/Unreported	33 (10.7%)	

*Note:* Compulsory program refers to the mandatory postgraduation service in underserved areas, specific to Iran. IRR refers to Iranian Rial; values are presented in millions.

Regarding the main study variables, the mean moral injury score was 41.12 ± 10.57, the mean burnout score was 90.43 ± 24.42, and the mean professional communication score was 129.58 ± 10.30. In terms of professional communication levels, 56.2% of nurses reported a moderate level (scores 114–133), 39.1% reported a strong level (scores 134–153), and 4.7% reported a poor level (scores 93–113).

Table 2 presents the association between demographic and occupational characteristics and the study variables. Professional communication was significantly higher among older nurses (*r* = 0.238, *p* < 0.001), married nurses (134.5 ± 8.6 vs. 126.2 ± 10.0, *p* < 0.001), and those working morning shifts (146.2 ± 3.1 vs. 129.4 ± 10.0 in rotating shifts, *p* < 0.001). Nurses who had received ethics training also reported higher professional communication (134.8 ± 7.6 vs. 128.6 ± 10.5, *p* < 0.001).

**TABLE 2:** Associations of professional communication, job burnout, and moral injury with participants’ characteristics.

Variable		Moral injury mean ± SD	*p* value	Job burnout mean ± SD	*p* value	Professional communication mean ± SD	*p* value
Age		Pearson’s *r* = −0.138	0.016^∗^	Pearson’s *r* = −0.145	0.019^∗^	Pearson’s *r* = 0.238	< 0.001^∗^
Gender	Female	42.90 ± 12.00	**< 0.001** ^∗∗^	94.40 ± 25.20	**< 0.001** ^∗∗^	130.50 ± 10.90	**0.046** ^∗∗^
	Male	38.70 ± 7.75		84.00 ± 21.69		127.90 ± 9.07	

Mean difference (95% confidence interval)		4.22 (1.85, 6.57)		10.38 (4.35, 16.40)		2.58 (0.051, 5.11)	
Effect size (95% confidence interval)		0.40 (0.17, 0.63)		0.43 (0.17, 0.68)		0.25 (0.004, 0.499)	
Marital status	Single	42.10 ± 11.40	**0.052** ^∗∗^	91.40 ± 21.80	**0.387** ^∗∗^	126.20 ± 10.00	**< 0.001** ^∗∗^
	Married	39.60 ± 8.92		88.60 ± 28.96		134.50 ± 8.59	

Mean difference (95% confidence interval)		2.41 (−0.021, 4.84)		2.78 (−3.54, 9.11)		−8.35 (−10.64, −6.06)	
Effect size (95% confidence interval)		0.22 (−0.0025, 0.46)		0.11 (−0.14, 0.37)		−0.88 (−1.14, −0.62)	
Employment type	Rasmi	38.50 ± 9.48	**< 0.001** ^∗∗∗^	88.80 ± 21.97	**0.328** ^∗∗∗^	127.50 ± 8.72	**< 0.001** ^∗∗∗^
	Peymani	46.00 ± 0.00		76.00 ± 0.00		132.00 ± 0.00	
	Gharardadi	45.30 ± 7.59		87.20 ± 36.33		139.70 ± 4.20	
	Tarhi	42.30 ± 11.80		93.10 ± 23.02		128.20 ± 11.46	

Tukey post hoc test		Official—Temporary	**0.002**	—	—	Official—Temporary	**< 0.001**
		Official—Compulsory	**0.017**			Compulsory—Temporary	**< 0.001**

Effect size (95% confidence interval)		0.054 (0.011, 0.103)		0.013 (0.000, 0.043)		0.164 (0.085, 0.236)	
Education	Associate degree	34.60 ± 2.76	**0.047** ^∗∗^	73.30 ± 27.97	**0.023** ^∗∗^	134.70 ± 2.00	**0.110** ^∗∗^
	Bachelor’s degree	41.30 ± 10.70		91.10 ± 24.10		129.40 ± 10.40	

Mean difference (95% confidence interval)		−6.75 (−13.40, −0.08)		−17.82 (−33.21, −2.43)		5.31 (−1.20, 11.83)	
Effect size (95% confidence interval)		−0.64 (−1.32, 0.06)		−0.73 (−1.43, −0.004)		0.51 (−0.16, 1.17)	
Experience (years)	1	43.70 ± 12.35	**0.076** ^∗∗∗^	87.10 ± 24.01	**0.016** ^∗∗∗^	123.40 ± 8.42	**< 0.001** ^∗∗∗^
	1–5	40.40 ± 8.70		96.90 ± 20.40		131.20 ± 10.82	
	5–10	39.50 ± 10.61		83.00 ± 16.12		125.90 ± 11.73	
	> 10	40.20 ± 10.36		89.50 ± 30.09		133.90 ± 7.97	

Tukey post hoc test		—	—	1–5 years‐5–10 years	**0.035**	1 year to 1–5 years	**< 0.001**
						1–5 years to 5–10 years	**0.049**
						1 year to > 10 years	**< 0.001**
						5–10 years to > 10 years	**< 0.001**

Effect size (95% confidence interval)		0.022 (0.00, 0.057)		0.039 (0.001, 0.087)		0.175 (0.09, 0.24)	
Shift type	Morning	46.75 ± 5.17	**0.272** ^∗∗∗^	60.25 ± 7.24	< 0.001^∗∗∗^	146.25 ± 3.10	**< 0.001** ^∗∗∗^
	Evening	39.00 ±0 .00		55.00 ±0 .00		118.00 ±0 .00	
	Rotating	41.03 ± 10.78		92.59 ± 23.63		129.42 ± 9.98	

Tukey’s post hoc test		—	—	Morning—rotating	< 0.001	Morning—evening	**< 0.001**
				Evening—rotating	< 0.001	Morning—rotating	**< 0.001**
						Evening—rotating	**0.003**

Effect size (95% confidence interval)		0.009 (0.00, 0.03)		0.12 (0.05, 0.19)		0.11 (0.04, 0.18)	
Monthly shifts (number)		Pearson’s *r* = −0.164	**0.004** ^∗^	Pearson’s *r* =	0.228^∗^	Pearson’s *r* =	**0.506** ^∗^
Monthly scrub hours		Pearson’s *r* = 0.019	**0.737** ^∗^	Pearson’s *r* = 0.122	0.049^∗^	Pearson’s *r* = −0.134	**0.026** ^∗^
Monthly circulator hours		Pearson’s *r* = −0.130	**0.023** ^∗^	Pearson’s *r* = −0.045	0.465^∗^	Pearson’s *r* = −0.052	**0.387** ^∗^
Overtime Hours Per Month		Pearson’s *r* = −0.030	**0.605** ^∗^	Pearson’s *r* = 0.215	< 0.001^∗^	Pearson’s *r* = −0.082	**0.174** ^∗^

Income (million IRR)	< 10	31.00 ± 0.00	**0.245** ^∗∗∗^	107.00 ± 0.00	0.492^∗∗∗^	135.00 ± 0.00	**0.045** ^∗∗∗^
	10–20	41.20 ± 11.00		90.20 ± 25.07		129.00 ± 10.32	
	> 20	41.60 ± 4.72		91.00 ± 19.61		133.80 ± 9.78	

Tukey’s post hoc test		—	—	—	—	10–20 million to 20‐30 million	**0.049**
Effect size (95% confidence interval)		0.009 (0.00, 0.037)		0.006 (0.00, 0.03)		0.023 (0.00, 0.06)	
Severe emotional trauma/loss (past 6 months)	Yes	39.93 ± 8.61	**0.124** ^∗∗^	96.75 ± 17.14	0.001^∗∗^	126.65 ± 9.58	**0.012** ^∗∗^
	No	41.51 ± 11.12		88.55 ± 25.95		130.68 ± 10.37	

Mean difference (95% confidence interval)		−1.58 (−4.34, 1.18)		8.20 (1.18, 15.23)		−4.03 (−6.74, −1.32)	
Effect size (95% confidence interval)		−0.14 (−0.41, 0.11)		0.33 (0.04, 0.63)		−0.39 (−0.66, −0.12)	
Ethics training provided at workplace	Yes	38.79 ± 10.19	**0.099** ^∗∗^	83.32 ± 17.54	**0.126** ^∗∗^	134.84 ± 7.57	**< 0.001** ^∗∗^
	No	41.55 ± 10.60		91.20 ± 24.95		128.60 ± 10.46	

Mean difference (95% confidence interval)		−2.76 (−6.05, 0.52)		−7.88 (−17.97, 2.21)		6.24 (2.94, 9.52)	
Effect size (95% confidence interval)		−0.26 (−0.57, 0.05)		−0.32 (−0.74, 0.10)		0.61 (0.26, 0.96)	

^∗^Pearson correlation coefficients.

^∗∗^Independent samples *t*‐test (effect size: Cohen’s d).

^∗∗∗^One‐way ANOVA (effect size: eta‐squared).

Burnout was higher among female nurses (94.4 ± 25.2 vs. 84.0 ± 21.7 in males, *p* < 0.001), female and less‐experienced nurses (96.9 ± 20.4 in those with 1–5 years of experience vs. 83.0 ± 16.1 in those with 5–10 years of experience, *p* = 0.035), and nurses working rotating shifts (92.6 ± 23.6, *p* < 0.001). Burnout was also positively correlated with the number of monthly shifts (*r* = 0.228, *p* = 0.004) and overtime hours (*r* = 0.215, *p* < 0.001).

Moral injury was higher among female nurses (42.9 ± 12.0 vs. 38.7 ± 7.8 in males, *p* < 0.001), contractual staff (45.3 ± 7.6, *p* < 0.001), and less‐experienced nurses (43.7 ± 12.3 in those with < 1 year of experience vs. 40.2 ± 10.4 in those with > 10 years of experience, *p* < 0.001). Moral injury was inversely correlated with age (*r* = −0.138, *p* = 0.016) and circulator hours (*r* = −0.130, *p* = 0.023). In addition, participants who had recently experienced emotional trauma reported lower professional communication (126.7 ± 9.6 vs. 130.7 ± 10.4, *p* = 0.012) and higher burnout (96.8 ± 17.1 vs. 88.6 ± 26.0, *p* = 0.001).

As shown in Table 3, professional communication was not significantly correlated with either moral injury (*r* = 0.086, *p* = 0.153) or burnout (*r* = −0.047, *p* = 0.461). By contrast, moral injury was positively correlated with burnout (*r* = 0.286, *p* < 0.001).

**TABLE 3:** Pearson correlation coefficients among professional communication, moral injury, and job burnout.

Variable	Moral injury	Job burnout	Professional communication
Moral injury	1		
Job burnout	*r* = 0.286^∗∗^	1	
Professional communication	*r* = 0.086	*r* = −0.047	1

Table 4 presents the results of the multiple linear regression analysis. To assess multicollinearity, variance inflation factor (VIF) values were examined. All VIF values were well below the critical threshold of 10, ranging from 1.05 to 1.21, indicating no evidence of problematic multicollinearity. Higher professional communication was significantly associated with lower burnout (*B* = −0.07, *p* = 0.001) and higher moral injury (*B* = 0.20, *p* < 0.001). Married nurses and those with longer work experience also reported higher professional communication. Among employment categories, temporary and compulsory staff scored significantly higher than official staff. In contrast, evening shifts (*B* = −23.07, *p* < 0.001) and rotating shifts (*B* = −13.08, *p* < 0.001) were associated with lower professional communication. Both the number of monthly shifts (*B* = 0.23, *p* = 0.009) and overtime hours (*B* = 0.05, *p* = 0.004) showed positive association with professional communication. Overall, the regression model explained 51.9% of the variance in professional communication (Adjusted *R* ^2^ = 0.492, *p* < 0.001), indicating that the included variables were important correlates in this clinical context.

**TABLE 4:** Multiple linear regression predicting professional communication.

				**95% confidence interval**		**t**	***p* value**
**Predictor**		**Estimate**	**SE**	**Lower**	**Upper**		

Intercept^a^		113.87	5.70	102.63	125.11	19.95	< 0.001
Moral injury		0.20	0.05	0.095	0.31	3.68	< 0.001
Job burnout		−0.07	0.023	−0.122	−0.029	−3.21	0.001
Marital status	Married (single: ref)	3.81	1.26	1.32	6.31	3.019	0.003
Employment type	Contractual (official: ref)	4.57	4.91	−5.10	14.25	0.93	0.353
	Temporary (official: ref)	6.85	1.81	3.28	10.43	3.78	< 0.001
	Compulsory (official: ref)	9.51	1.93	5.69	13.33	4.90	< 0.001

Work experience	1–5 years (1 year: ref)	16.09	1.780	12.58	19.60	9.04	< 0.001
	5–10 years (1 year: ref)	11.24	2.82	5.68	16.80	3.98	< 0.001
	> 10 years (1 year: ref)	18.80	2.66	13.55	24.06	7.04	< 0.001

Shift type	Evening (morning: ref)	−23.07	4.37	−31.69	−14.46	−5.27	< 0.001
	Rotating (morning: ref)	−13.08	3.13	−19.25	−6.91	−4.17	< 0.001

Monthly shifts (number)		0.23	0.08	0.06	0.41	2.65	0.009
Monthly circulator hours		0.05	0.01	0.01	0.08	2.91	0.004

*Note:* Model summary: *R* ^2^ = 0.519; Adjusted *R* ^2^ = 0.492; overall model: *F*(13, 293) = 24.32, *p* < 0.001. A *p* value < 0.05 was considered statistically significant. Reference categories: marital status (single), employment type (official), work experience (< 1 year), shift type (morning).

Abbreviations: CI = confidence interval; SE, standard error.

## 5. Discussion

This study examined the association of moral injury and job burnout with professional communication among OR nurses in Southern Iran. The results demonstrated a significant positive association between moral injury and burnout. Contrary to expectations, the bivariate analysis did not identify a significant relationship between either moral injury or burnout and professional communication. However, in the multivariate regression model, moral injury emerged as a positive correlate of professional communication, whereas burnout showed a negative association. In addition, several demographic and occupational characteristics, including marital status, type of employment, work experience, and shift pattern, were significantly associated with the quality of professional communication.

The present study identified a significant positive association between moral injury and job burnout among OR nurses (*r* = 0.286, *p* < 0.001). This pattern can be interpreted through the Job Demands–Resources (JD‐R) model, which conceptualizes morally injurious experiences as exhausting job demands that may deplete psychological and emotional resources and increase vulnerability to burnout [22]. Similarly, Lazarus and Folkman’s Transactional Model of Stress suggests that when nurses encounter situations that threaten their ethical values, these experiences may be appraised as significant stressors, contributing to sustained occupational strain and fatigue [23].

This finding is consistent with much of the existing literature. Wu et al. [12], for example, reported that moral distress was directly associated with burnout among emergency nurses in China, with ethical climate and moral resilience mediating this relationship. Likewise, Lotfi‐Bejestani et al. [24] identified significant associations between burnout, moral distress, and perceptions of an unjust work environment among nurses in Iran. Recent regional studies in the Middle East also support this interpretation. Ayed et al. [25] and Batran et al. [26] reported that stressful work environments were associated with poorer professional quality of life and higher burnout among nurses in intensive care and neonatal care settings. During the COVID‐19 pandemic, Mantri et al. [27] also demonstrated that healthcare workers with higher levels of moral injury experienced greater burnout.

Nevertheless, some studies have yielded inconsistent findings. For instance, Galanis et al. [28] found that moral resilience may serve as a protective factor that mitigates the adverse effects of moral distress on burnout. Similarly, Aqtam et al. [29] highlighted the interplay between occupational stress and resilience in high‐acuity settings, suggesting that resilience‐building interventions may help buffer the psychological impact of moral stressors. Zakeri‐Afshar et al. [30] also reported no direct association between burnout and moral courage among OR nurses in Iran. These discrepancies may reflect differences in study populations, measurement instruments, organizational context, pandemic‐related conditions, or protective factors such as social support, moral resilience, and organizational justice.

Taken together, the present findings reinforce the broader evidence base suggesting that moral injury is an important correlate of burnout among OR nurses. However, the moderate correlation observed in this study (*r* = 0.286) indicates that other individual and organizational factors, including moral resilience, moral courage, and organizational support, may also contribute to nurses’ burnout experiences. From a practical perspective, this finding highlights the importance of organizational strategies that strengthen moral resilience, promote ethical support, and foster a supportive work climate.

One of the unexpected findings of this study was that moral injury was positively associated with professional communication in the multivariate regression model, although this association was not statistically significant in the bivariate correlation analysis. This finding should be interpreted cautiously. It should not be understood as evidence that moral injury improves professional communication or has a beneficial role in the workplace. Rather, the emergence of a positive adjusted association may reflect the influence of multivariate adjustment, shared variance with burnout, or suppression effects. In other words, after controlling for burnout and relevant demographic and occupational characteristics, the unique variance of moral injury may have captured a dimension related to moral sensitivity, ethical awareness, or professional advocacy. One possible explanation is that nurses who report higher moral injury may also have greater moral sensitivity, stronger awareness of ethical conflict, or greater involvement in ethically challenging clinical situations. In such situations, professional communication may function as a means of expressing concerns, seeking clarification, advocating for patients, or negotiating professional responsibilities. This interpretation is broadly consistent with Social Support Theory, which suggests that individuals experiencing stress may seek interpersonal support and professional dialog as coping resources [31]. It is also compatible with Conservation of Resources theory, which proposes that individuals attempt to conserve, protect, and restore psychological and social resources when exposed to stressful or resource‐threatening conditions [32].

Direct empirical evidence on the relationship between moral injury and professional communication remains limited. However, related studies may help contextualize this finding. For example, Li et al. [13, 33] reported that moral injury was associated with healthcare workers’ sense of mission and meaning at work, suggesting that morally injurious experiences may be closely connected to how professionals interpret and communicate ethical concerns in clinical practice. In addition, Dehghani et al. [34] found that communication skills training was associated with lower moral distress among nurses, indicating that communication may play a protective or regulatory role in ethically challenging situations.

This interpretation should be considered with caution, but it offers a clinically meaningful perspective on the role of moral injury in professional communication. In morally challenging situations, OR nurses may need to communicate more actively to clarify responsibilities, seek support from colleagues, advocate for patients, or negotiate ethical concerns within the surgical team. Therefore, the positive adjusted association observed in this study may reflect a form of ethically motivated communication rather than a beneficial effect of moral injury itself. From a nursing management perspective, this finding highlights the importance of creating psychologically safe environments in which nurses can express ethical concerns before these experiences progress into persistent moral distress, emotional withdrawal, or burnout. Practical strategies may include ethics debriefing sessions, supportive supervision, confidential ethics consultation pathways, and team‐based reflection after morally challenging events [35].

The results also showed that burnout was not significantly associated with professional communication in the bivariate correlation analysis (*r* = −0.047, *p* = 0.461), but it was significantly associated with lower professional communication in the multivariate regression model (*B* = −0.07, *p* = 0.001). At first glance, this pattern may appear unexpected, as burnout—characterized by emotional exhaustion, reduced personal efficacy, and depersonalization—is theoretically expected to impair interpersonal interactions and collaboration. In the OR context, however, professional obligations and patient safety demands may require nurses to maintain essential communication even when experiencing burnout.

From the perspective of the JD‐R model, intense job demands are closely linked to burnout, whereas supportive resources such as team cohesion, solidarity, and collegial support may buffer its negative association with communication [22]. Similarly, the Burnout–Engagement Model suggests that nurses may sustain professional engagement and communication despite burnout because of their ethical commitment to patients and sense of responsibility toward the surgical team [36].

Previous research supports this interpretation. Bayer and Öztürk [37], for example, found that although OR nurses reported high levels of emotional exhaustion and depersonalization, their communication skills within the team remained relatively strong. In contrast, qualitative evidence from the Iranian context suggests that prolonged burnout caused by organizational pressures and negative interpersonal interactions may gradually erode professional communication over time [38]**.** These divergent findings may reflect differences in cultural context, organizational conditions, and coping strategies adopted by surgical teams.

The relationship between burnout and professional communication among OR nurses therefore appears to be complex and multifactorial. The nonnegotiable demands of teamwork in the OR may partly mask or mitigate the adverse association of burnout with communication quality. These findings underscore the need for nursing managers to implement burnout‐reduction programs while also strengthening team‐based support and communication skills, so that professional interactions can be preserved even under stressful clinical conditions.

The findings further revealed that certain individual and occupational characteristics were associated with nurses’ experiences of moral injury, burnout, and professional communication. Female nurses reported higher levels of burnout, whereas married nurses demonstrated stronger professional communication. Nurses with a bachelor’s degree experienced higher levels of moral injury and burnout compared with those with higher qualifications. Contractual nurses reported stronger professional communication but also higher levels of moral injury. Longer work experience was associated with better communication skills, while early career stages were linked to increased burnout. In addition, rotating shifts were associated with higher burnout, which is consistent with previous evidence showing that prolonged and irregular shifts are associated with greater burnout and lower job satisfaction [39].

### 5.1. Implications for Nursing Management

The findings of this study have several implications for nursing management in OR settings. First, professional communication should be approached as a managerial and organizational priority rather than solely as an individual skill. The observed association between lower burnout and better professional communication suggests that strategies aimed at reducing burnout may also support more effective communication among OR nurses. Nursing managers should therefore consider interventions such as workload monitoring, fair shift allocation, adequate staffing, structured rest periods, and access to psychological support services, particularly for nurses working evening or rotating shifts.

Second, the higher levels of burnout and moral injury observed among female nurses and less‐experienced staff suggest the need for targeted but nonstigmatizing support strategies. Newly employed and less‐experienced OR nurses may benefit from structured mentorship, communication‐skills training, ethics education, and supervised transition‐to‐practice programs. Similarly, managers should ensure that support systems are accessible to all nurses while remaining sensitive to groups who may report higher psychological strain. The finding that nurses who had received ethics training reported better professional communication further supports the value of integrating ethics‐based communication training into continuing professional development programs.

Third, the positive association between moral injury and professional communication in the regression model should be interpreted cautiously. This finding does not suggest that moral injury is beneficial or should be tolerated in the workplace. Rather, it may indicate that nurses with greater moral sensitivity or stronger awareness of ethical conflict are more likely to engage in communication about patient care, professional responsibilities, or ethically challenging situations when other factors are controlled. From a management perspective, this highlights the importance of creating psychologically safe environments in which nurses can express ethical concerns without fear of blame, retaliation, or professional isolation. Ethics debriefing sessions, confidential consultation pathways, supportive leadership responses, and team‐based reflection after morally challenging events may help transform ethical distress into constructive communication rather than silence, withdrawal, or burnout.

Finally, although monthly shifts and overtime hours showed positive associations with professional communication in the regression model, these findings should not be interpreted as evidence that increasing workload improves communication. They may reflect greater clinical exposure, role familiarity, or the presence of more experienced nurses in heavier workload categories. Therefore, managers should avoid using workload intensification as a strategy for improving communication. Instead, efforts should focus on maintaining safe staffing, supporting communication during high‐pressure shifts, and reducing organizational conditions that contribute to burnout and moral strain.

### 5.2. Strength and Limitations of the Work

A major strength of this study is its simultaneous examination of three relatively underexplored variables—moral injury, burnout, and professional communication—among OR nurses, thereby addressing an important gap in the literature. In addition, sampling from multiple tertiary surgical centers and the use of culturally adapted instruments enhanced the contextual relevance of the findings for Iranian nurses. However, several limitations should be acknowledged. The cross‐sectional design precludes causal inference, and the reliance on self‐report measures may have introduced social desirability bias. In addition, the exclusion of participants with a history of psychiatric treatment or psychotropic medication use may have introduced selection bias by potentially excluding individuals experiencing more severe moral injury or occupational burnout. Finally, the restriction of the sample to a single geographical region limits the generalizability of the findings.

### 5.3. Recommendations for Further Research

Future research should use longitudinal and interventional designs to clarify the pathways linking moral injury, burnout, and professional communication. Moreover, examining the mediating roles of organizational support, ethical leadership, psychological safety, and professional empathy may provide a deeper insight into how these factors interact to influence communication quality in high‐stress clinical settings.

## 6. Conclusion

This study showed that moral injury and burnout are associated with professional communication among OR nurses. Moral injury was positively associated with burnout and, unexpectedly, showed a positive adjusted association with professional communication in the multivariate regression model. This finding should be interpreted cautiously and may reflect ethically motivated communication, moral sensitivity, or professional advocacy rather than a beneficial effect of moral injury itself. Burnout, in contrast, was associated with lower professional communication after adjustment for demographic and occupational factors.

These findings suggest that nursing managers should consider integrated strategies to reduce burnout, support nurses facing morally challenging situations, and strengthen professional communication within surgical teams. Such strategies may include moral resilience training, workload management, shift schedule optimization, ethics debriefing, and team‐based communication training. Future longitudinal and qualitative studies are needed to clarify the direction and mechanisms of the relationships among moral injury, burnout, and professional communication.

## Author Contributions

All authors have met the criteria for authorship according to the International Committee of Medical Journal Editors (ICMJE). Specifically, all authors have made substantial contributions to the conception or design of the work or the acquisition, analysis, or interpretation of data. All authors have participated in drafting the article or revising it critically for important intellectual content.

Amirali Alizadeh, Zahra Maleki, Mahdiyeh Shahmoradi, and Fatemeh Vizeshfar were involved in study design. Mahdiyeh Shahmoradi and Fatemeh Davodabadi were concerned with data collection. Zahra Maleki did data analysis. Amirali Alizadeh and Fatemeh Vizeshfar were responsible for study supervision. Amirali Alizadeh, Zahra Maleki, Fatemeh Davodabadi, and Fatemeh Vizeshfar were involved in manuscript writing. Amirali Alizadeh, Zahra Maleki, Fatemeh Davodabadi, Fatemeh Vizeshfar, and Mahdiyeh Shahmoradi did critical revisions for important intellectual content.

## Funding

This research received no specific grant from any funding agency in the public, commercial, or not‐for‐profit sectors.

## Disclosure

There is a statistician on the author team: Zahra Maleki. The authors affirm that the methods used in data analyses are suitably applied to their data within their study design and context, and the statistical findings have been implemented and interpreted correctly. The authors agree to take responsibility for ensuring that the choice of the statistical approach is appropriate and is conducted and interpreted correctly as a condition to submit to the journal. All authors gave final approval of the version to be published.

## Ethics Statement

The study protocol was approved by the Biomedical Research Ethics Committee of Shiraz University of Medical Sciences (IR.SUMS.NUMIMG.REC.1403.114‎). Written informed consent was obtained from all participants prior to their enrollment in the study.

## Conflicts of Interest

The authors declare no conflicts of interest.

## Data Availability

The datasets used and analyzed during the current study are available from the corresponding author upon reasonable request.
